# Src Dependent Pancreatic Acinar Injury Can Be Initiated Independent of an Increase in Cytosolic Calcium

**DOI:** 10.1371/journal.pone.0066471

**Published:** 2013-06-18

**Authors:** Vivek Mishra, Rachel Cline, Pawan Noel, Jenny Karlsson, Catherine J. Baty, Lidiya Orlichenko, Krutika Patel, Ram Narayan Trivedi, Sohail Z. Husain, Chathur Acharya, Chandra Durgampudi, Donna B. Stolz, Sarah Navina, Vijay P. Singh

**Affiliations:** 1 Department of Medicine, University of Pittsburgh, Pittsburgh, Pennsylvania, United States of America; 2 Department of Cell Biology and Physiology, University of Pittsburgh, Pittsburgh, Pennsylvania, United States of America; 3 Department of Pediatrics, University of Pittsburgh Medical Center, Pittsburgh, Pennsylvania, United States of America; 4 Department of Medicine, University of Pittsburgh Medical Center, Passavant, Pennsylvania, United States of America; 5 Department of Pathology, University of Pittsburgh, Pittsburgh, Pennsylvania, United States of America; University of Houston, United States of America

## Abstract

Several deleterious intra-acinar phenomena are simultaneously triggered on initiating acute pancreatitis. These culminate in acinar injury or inflammatory mediator generation *in vitro* and parenchymal damage *in vivo*. Supraphysiologic caerulein is one such initiator which simultaneously activates numerous signaling pathways including non-receptor tyrosine kinases such as of the Src family. It also causes a sustained increase in cytosolic calcium- a player thought to be crucial in regulating deleterious phenomena. We have shown Src to be involved in caerulein induced actin remodeling, and caerulein induced changes in the Golgi and post-Golgi trafficking to be involved in trypsinogen activation, which initiates acinar cell injury. However, it remains unclear whether an increase in cytosolic calcium is necessary to initiate acinar injury or if injury can be initiated at basal cytosolic calcium levels by an alternate pathway. To study the interplay between tyrosine kinase signaling and calcium, we treated mouse pancreatic acinar cells with the tyrosine phosphatase inhibitor pervanadate. We studied the effect of the clinically used Src inhibitor Dasatinib (BMS-354825) on pervanadate or caerulein induced changes in Src activation, trypsinogen activation, cell injury, upstream cytosolic calcium, actin and Golgi morphology. Pervanadate, like supraphysiologic caerulein, induced Src activation, redistribution of the F-actin from its normal location in the sub-apical area to the basolateral areas, and caused antegrade fragmentation of the Golgi. These changes, like those induced by supraphysiologic caerulein, were associated with trypsinogen activation and acinar injury, all of which were prevented by Dasatinib. Interestingly, however, pervanadate did not cause an increase in cytosolic calcium, and the caerulein induced increase in cytosolic calcium was not affected by Dasatinib. These findings suggest that intra-acinar deleterious phenomena may be initiated independent of an increase in cytosolic calcium. Other players resulting in acinar injury along with the Src family of tyrosine kinases remain to be explored.

## Introduction

Pancreatitis is initiated by numerous insults [1]. The most commonly used model to study pancreatitis in rodents is the caerulein model. Caerulein, an octa-peptide analog of the hormone cholecystokinin, at supraphysiologic concentrations initiates multiple signaling cascades simultaneously, which eventually culminate in cell death and inflammatory mediator generation [2]–[6]. The commonly studied upstream signaling mechanisms include the activation of Src [7], protein kinase C isoforms [8], [9], calcium signaling [10]–[12], the calcium dependent protein kinase Pyk2 [13], [14], PI3 Kinases [15]–[17], MAP kinases [18]–[20], ERK [21] etc. These regulate phenomena such as actin reorganization [22]–[24], caspase activation [5], [6], transcription factor activation such as AP-1 [2], translocation of p65 unit of NF-κB to the nucleus [25], vesicular trafficking such as from the Golgi [26], trypsinogen activation [26], mitochondrial depolarization [5], [27], reactive oxygen species formation [27]- eventually initiating a mode of cell death or the generation of inflammatory mediators.

Calcium is thought to be an essential player in cell death and proinflammatory pathways. Studies reducing intracellular or extracellular calcium concentrations using various methods [28], such as calcium chelators (e.g. BAPTA –AM or EGTA) [29] or antagonists to intracellular receptors upstream of the release of calcium (e.g. the inositol triphosphate, or ryanodine receptors) [30], [31], have supported its role in these pathways. The increase in intracellular calcium is commonly thought to be “essential but not sufficient” for the initiation of these pathways since calcium chelators do prevent certain crucial steps (e.g. trypsinogen activation, NF-κB activation) [2], [32], but agents that increase intracellular calcium alone (e.g. ionomycin or thapsigargin) have been ineffective in activating proinflammatory or cell death phenomena in most studies [2], [28], [32].

Acinar cells express numerous members of the Src family of tyrosine kinases [7], [22], [33]–[36], the roles of which in caerulein induced outcomes is currently being explored. Phenomena involving Src include basolateral reorganization of actin, which is dependent on cortactin phosphorylation perhaps via the Src family member Yes [7], [22]. This results in blebbing and acinar injury. Recently c-Src has been implicated in vesiculation of the Golgi and transit of proteins through the Golgi cisternae have been inhibited by mutant dynamin that cannot be phosphorylated by Src [37]. Our recent studies have supported the antegrade vesiculation of the Golgi in pancreatic acinar cells to be associated with pro-cathepsin B processing and interference with this to result in prevention of caerulein induced trypsinogen activation [26].

While Src family members are activated by various hormones that act via cell surface receptors, the agonists that activate one family member may not activate another [13], [36]. Moreover some ligands like bombesin and carbamylcholine, which activate certain family members (e.g. Yes) [13] also increase intracellular calcium. The calcium dependent tyrosine kinase Pyk2 is also activated by stimuli such as supramaximal caerulein [14]. We aimed to explore the cell biologic and phenotypic outcomes of globally activating tyrosine kinases, if this is associated with an increase in intracellular calcium and to learn whether Src may play a role in these outcomes. To globally activate tyrosine kinase signaling in a non-receptor mediated manner, we treated freshly prepared acinar cells (which are able to generate trypsin, unlike those after overnight culture) with the tyrosine phosphatase inhibitor pervanadate, and studied its effects in the presence and absence of the Src inhibitor Dasatinib. Dasatinib (BMS-354825) [38] is a highly specific inhibitor of the Src-Abl family of tyrosine kinase which is approved for human use [39]–[41]. Previously we have shown the pyrazolo-pyrimidine Src inhibitor PP2 and SU6656 to interfere with caerulein induced Src mediated actin reorganization [7]. We also compared the effects of pervanadate to those of supraphysiologic caerulein.

Interestingly, while pervanadate mimicked the biochemical effects of supraphysiologic caerulein in initiating the morphological changes and deleterious phenotypic outcomes in acinar cells, its effects were independent of an increase in cytosolic calcium. Further, Dasatinib inhibited Src and prevented these outcomes without affecting calcium signaling. These findings support the existence of calcium independent mechanisms by which injury may be initiated in acinar cells.

## Materials and Methods

### Animals

CD-1/ICR mice were purchased from Charles River Laboratories (Wilmington, MA). Mice were housed with a 12-h light/dark cycle, at temperatures from 21–25C, were fed standard laboratory chow, and allowed to drink ad libitum. Caerulein was purchased from Bachem (King of Prussia, PA). Dasatinib was purchased from LC labs (Woburn, MA). All other reagents and chemicals were purchased from Sigma (St. Louis, MO). All experimental protocols were approved by the Institutional Animal Use Committee of the University of Pittsburgh (Pittsburgh, PA).

### Preparation and the Use of Acini

This procedure was carried out as previously described [42], [43]. Fresh acini were used for all assays. For treatment with inhibitors, acini were incubated for 1 hour with Dasatinib (10 mM stock in DMSO, final concentration 10 µM) prior to adding the stimulus (100 nM caerulein; CER, or 100 µM pervanadate; PV). Acinar viability before use was >95%, as indicated by trypan blue exclusion.

### Immunofluorescence Studies

**Figure 2:**
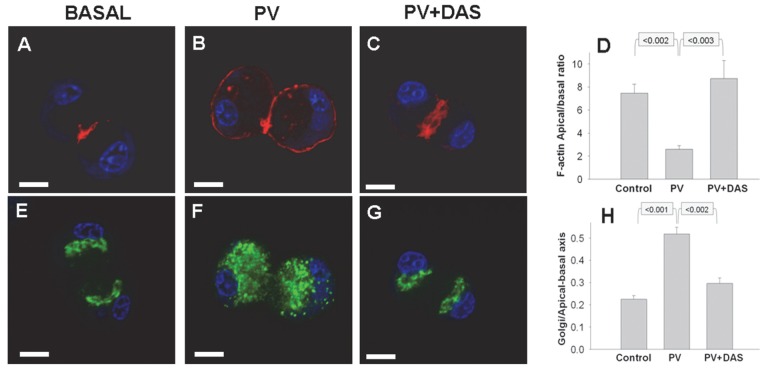
Pervanadate induced F-actin reorganization and antegrade Golgi fragmentation is inhibited by Dasatinib. Fluorescence imaging of acini under basal conditions (Basal, A, E), after stimulation with 100 µM pervanadate (PV) for 10 minutes (B,F) or pervanadate treated acini in the presence of Dasatinib (PV+DAS, C,G) showing F-actin (Red), the Golgi marker GM130 (green) and nuclei (Blue). Under basal conditions F-actin (A) is enriched in the sub-apical area, and the stacks of Golgi (E) in the supranuclear area occupying <25% of the apical-basal axis length (H). 100 µM pervanadate within 10 minutes reorganizes the F-actin to the basal surface (B) and induces antegrade fragmentation of the Golgi (F). The pervanadate induced changes in F-actin (C) and Golgi (G) are prevented in acini pre-incubated with 10 µM Dasatinib. Quantification of integrated density of apical to basal ratios of F-actin staining (D) and ratio of Golgi length in the apical-basal axis to that of the acinar cell (H) from 3 different experiments are shown. The measurement bar (left lower corner) depicts 10 µm.

These were done on acinar cells filtered through a 70 micron mesh treated as described in the results section and in the legend of Figure 2, and were processed as previously described [7]. Briefly, these were fixed with 2% paraformaldehyde, permeabilized, blocked with 5% normal goat serum, and exposed to an antibody against GM130 (1∶500, BD biosciences, San Jose, CA) for 1 hour. After 3 washes, goat anti-rabbit Alexa 488 (Invitrogen, Carlsbad, CA.), and DRAQ5 (1∶1000) for nuclear staining, or Rhodamine phalloidin [7], [44] were added for 1 hour. After washing, slides were mounted (fluormount, Sigma, St. Louis, MO) and imaged on a Zeiss Meta (LSM510) confocal microscope using a 63× lens and 1 micron thick optical sections. Images were processed as described previously [7] and analyzed using Adobe Photoshop CS4. Quantification was done for cells parallel to the coverslip. For F-actin quantification, as described by Torgerson and McNiven [24], a box was placed to cover entire apical or basolateral domains or a background area, and the integrated density from each of these was quantified. The background density was subtracted from that of the apical or basolateral domains and the results were depicted as an apical to basal ratio. For measuring antegrade extension of the Golgi, the pixel length of the apical to basal axis of the cell, and the pixel length of the Golgi over which this line passed were measured. Results were calculated as length of the Golgi as a ratio of apical-basal axis. 25–30 cells were quantified for each condition from 3 different experiments. The 3 groups were compared using a one way ANOVA.

### Trypsin Assay

This was done by the method of Kawabata et al [45] as described previously [26]. Briefly, after treatment with CER 100 nM, PV 100 µM, CER+DAS (10 µM) and PV+DAS (10 µM) for 30 min and washed with MOPS buffer [46] to stop stimulation. Cell pellets were homogenized in MOPS containing buffer. Trypsin activity was measured by a kinetic assay quantifying cleavage of the substrate (Boc-Gln-Ala-Arg-MCA, trypsin 3135; Peptides International, Inc., Louisville, KY) in the buffer [50 mM Tris (pH 8.1), 150 mM NaCl, 1 mM CaCl2, 0.01% BSA] flurometrically [(Versa FluorTM, Biorad) (Excitation 380-nm, Emission 440-nm] and quantified as arbitrary units per microgram of DNA in the homogenate of the acini sample and expressed as percentage maximal.

### Lactate Dehydrogenase Assay

Acinar cell injury was measured using a cytotoxicity assay for lactate dehydrogenase (LDH) leakage (Roche Applied Sciences, Indianapolis, IN) after 4 hours of treatment. Absorbance was measured at 490 nm and background at 690 nm, 15 min after stopping the enzyme reaction. Results were expressed as percent of total LDH (after lysis with 1% Triton-X100).

### Intracellular Calcium Imaging

Acinar cells alone or after treatment with Dasatinib (10 µM) as above were loaded with Fura-2AM as described previously [47], coated on glass bottom culture dishes (MatTek corporation, Ashland, MA) and imaged on a temperature-controlled motorized stage of an Olympus IX81 inverted microscope (Melville, NY) with a 20× (0.70 NA) objective and a QImaging Retiga EXi CCD camera (QImaging, Burnaby, Canada). Baseline images were taken, and cells with extremely bright or dim fluorescence were omitted. Caerulein (100 nM) or PV (100 µM) were added, and cytosolic calcium levels were determined by alternate excitation at 340 nm and 380 nm, measuring emission at 510 nm. Pre- and post-images using differential interference contrast were obtained to demonstrate appropriate cell morphology. Image acquisition was with the MetaMorph 7.5 Imaging System using the MetaMorph 6.3 software. The 340/380 emission ratio was averaged for 7–25 acini per field, with background subtraction for each experiment.

### Immunoprecipitation and Western Blotting

Isolated acini were stimulated with CER 100 nM, PV 100 µM, CER+DAS (10 µM) and PV+DAS (10 µM) for various times and washed with ice-cold PBS to stop stimulation. Acini were homogenized in lysis buffer containing various protease inhibitors (Complete, EDTA Free; Roche, Mannheim, Germany). Lysates were used for Immunoprecipitation after protein estimation with a Pierce protein assay kit (Thermo Fisher Scientific, Rockford, IL). In this case, lysates (1 mg/ml) were incubated with 5 µg/ml anti-Src primary antibody (SC-18; Santa Cruz Biotechnology, Inc., CA) for 2 h at 4°C, followed by addition of 4 mg of protein-A beads for 1 h in the same buffer. The beads were then washed three times, boiled in 1× Laemmli sample buffer, and analyzed by Western blot. Membranes for western blots were incubated with primary antibody [p-Src (Y-416); (1∶500) (Cell Signaling, MA)] and then probed with horseradish peroxidase-labeled goat anti-rabbit IgG (Sigma, St. Louis, MO). To visualize the band intensity on membrane, autoradiography was performed by using ECL plus Western Blot Detection Kit (Amersham GE Health care, Buckinghamshire, UK). Intensity of bands was quantified by scanning the film, storing it as a TIFF file and measuring the integrated density of each band in Adobe Photoshop CS4 after subtracting the back ground. Active Src levels (PY-416) were normalized to total Src (SC-18) for loading. Results were expressed as fold change over basal.

### Statistical Analysis

Data depicted is from at least 3 different experiments. Images shown are representative images from these and each parameter is shown as mean ± SEM. Pair wise comparisons were done using the Student’s T test for normally distributed data and the Mann-Whitney test for data without a normal distribution. Multiple groups were compared using one way ANOVA. A *p* value of <0.05 was regarded as significant.

## Results

### Pervanadate Induces Src Activation Which is Prevented by Dasatinib

**Figure 1:**
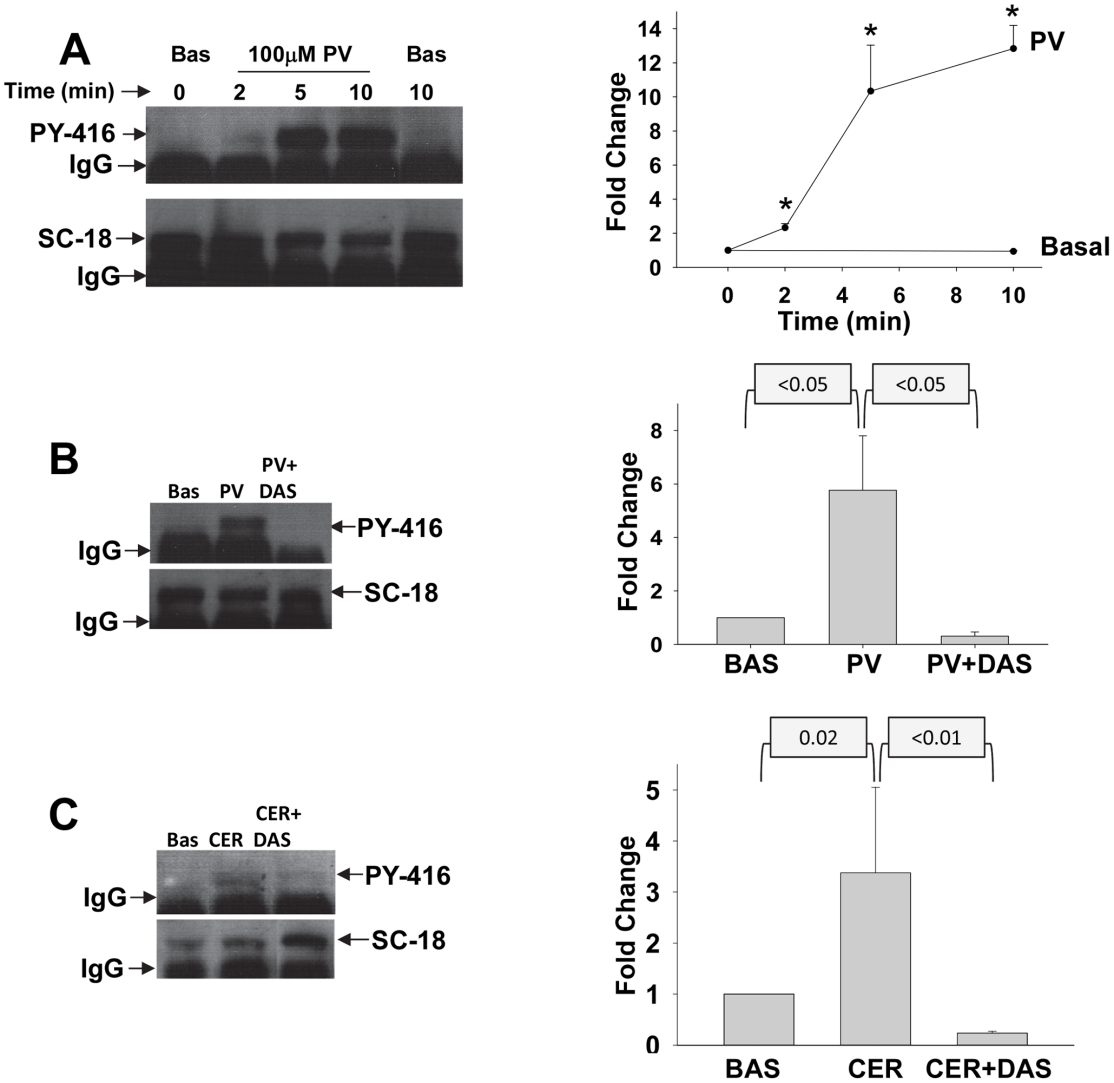
Src is activated by pervanadate and supramaximal Caerulein. Western-blot of immunoprecipitated Src after treatment of acini with 100 µM pervanadate (PV) for various times (A), 2 minutes of 100 µM pervanadate with or without pre-incubation with 10 µM Dasatinib (DAS) (B) or 100 nM caerulein (CER) with or without pre-incubation with 10 µM Dasatinib (Das) (C). These were blotted for antibodies to Src PY416 (i.e. active Src, upper panel), and then stripped and blotted for Total Src (SC-18, Lower panel). Corresponding graphs shown on the right show active Src levels (PY-416) as a ratio to total Src (SC-18) depicted as fold change over basal (BAS). Each data point was calculated from 3 or more experiments. For figure 1A, the graph depicts fold increase over BAS at the time of adding the stimulus and the asterisks in the graph depict a *p* value of ≤0.02. *p* values for the graphs corresponding to figure 1B, C are mentioned above these.

Addition of the tyrosine phosphatase inhibitor pervanadate (PV) at the commonly used concentration of 100 µM resulted in rapid activation of Src as seen by an increase in its phosphorylation at Y416 on western blotting of Src immuno-precipitates (Figure 1A). This was sustained over 10 minutes. This increase in Y416 phosphorylation was similar to what we have previously noted with supramaximal caerulein, which induces cell injury [7]. Pervanadate induced activation of Src was prevented by the Src inhibitor Dasatinib as evidenced by a lack of increase in Y416 phosphorylation (Figure 1B). Likewise Dasatinib prevented 100 nM caerulein induced activation of Src (Figure 1C).

### Pervanadate Induces Basolateral F-actin Reorganization, Antegrade Golgi Fragmentation which is Prevented by Src Inhibition

Since pervanadate activates the Src family, we then studied F-actin localization, which we have previously shown is dependent on Src activation in pancreatic acinar cells [7]. Normally acinar cells have enrichment of F-actin (shown in red) in the sub-apical areas of acinar cells (Figure 2A). Pervanadate (100 µM) caused reorganization of F-actin to the basolateral areas (Figure 2A&B) with a reduction in the apical to basal F-actin ratio (Figure 2D). This was dependent on Src as evidenced by prevention of this phenomenon by Dasatinib (Figure 2C, D). This phenomenon is very similar to caerulein induced actin reorganization [24], which is prevented by the Src inhibitor PP2 [7].

The Golgi in acinar cells (shown in green) is normally arranged as compact stacks in the supra-nuclear area (Figure 1E), the thickness of which (measured as apical-basal length) is normally less than 25% of the length of apical-basal axis of the cells (Figure 2H). We have recently shown caerulein to cause antegrade fragmentation of the Golgi in pancreatic acinar cells [26]. Recent studies have shown that Src regulates similar Golgi phenomena in other cells [37]. We therefore studied if Src activation by pervanadate may result in antegrade fragmentation of the Golgi. Indeed, pervanadate treatment for 10 minutes disrupted the Golgi stacks in an antegrade manner with the Golgi extending to 51.8±3% of the apical-basal axis (Figure 2F, H). This extension was prevented by Dasatinib (29.6±2.5%, *p*<0.002 Figure 2G, H).

### Pervanadate Induced Trypsinogen Activation and Acinar Injury is Dependent on Src Activation

**Figure 3:**
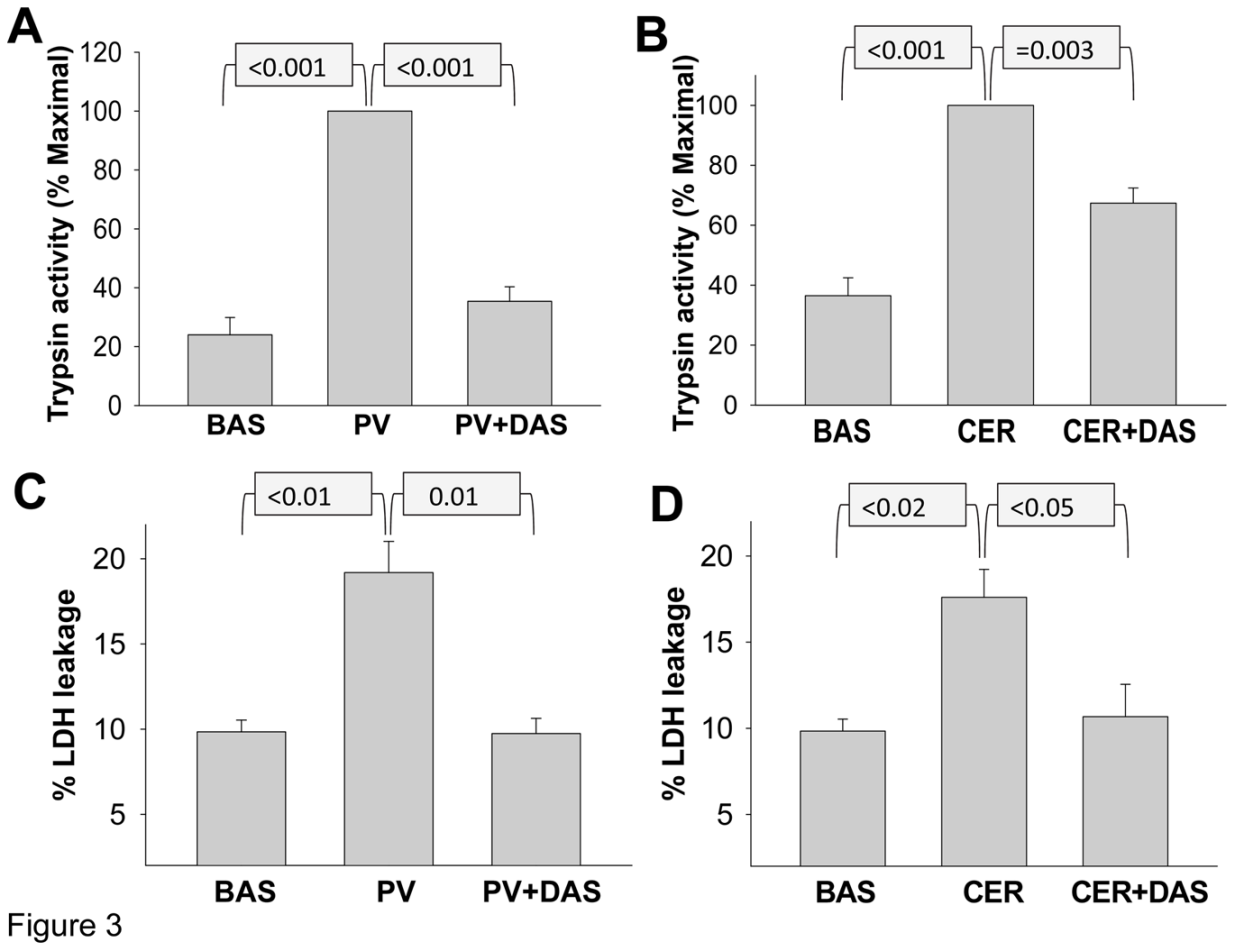
Dasatinib reduces pervanadate and caerulein induced trypsinogen activation and acinar cell injury. Trypsin activity is increased in cell homogenates from acini treated with 100 µM pervanadate (PV) (A), 100 nM caerulein (CER) (B) for 30 minutes. Lactate dehydrogenase (LDH) leakage is increased from acini treated with 100 µM pervanadate (PV) (C), 100 nM caerulein (D) for 4 hours. Preincubation with 10 µM Dasatinib (DAS) prevents these phenomena in response to both pervanadate and caerulein (A, B, C, D). BAS; Basal conditions. *p*-values mentioned in the figure were calculated using the Student’s t-test. Each bar representing mean ± SEM, was calculated from at least 3 different experiments.

We have recently shown that trypsinogen activation is regulated by post Golgi trafficking. We therefore studied if pervanadate treatment would result in trypsinogen activation. Pervanadate treatment of acini for 30 minutes resulted in a 4.2 fold increase in trypsinogen activation in acinar homogenates compared to acini under basal conditions (Figure 3A). This was significantly reduced by inhibiting Src with Dasatinib. Similarly, supramaximal (100 nM) caerulein induced trypsinogen activation (2.7 fold basal, Figure 3B) was significantly reduced by Dasatinib. Therefore Src activation seems to regulate trypsinogen activation.

Since intra-acinar protease activation, and actin reorganization are thought to be involved in acinar cell injury, we therefore studied if LDH leakage, a marker of acinar injury is affected by changes affecting trypsinogen activation. Indeed incubation of acinar cells with pervanadate resulted in an increase in LDH leakage compared to the acini incubated under basal conditions (19.2 vs 9.8 percent *P*<0.01, Figure 3C). This was similar in extent to the amount of LDH leakage induced by supramaximal (100 nM) caerulein (17.6 vs 9.8 percent *P*<0.02, Figure 3D). Src inhibition with Dasatinib resulted in a reduction in both pervanadate and caerulein induced acinar injury. Therefore Src seems to regulate both deleterious phenomena, i.e. intra-acinar protease activation and actin reorganization, consequently regulating acinar injury.

### Pervanadate Induced Phenomena are Independent of an Increase in Cytosolic Calcium

**Figure 4:**
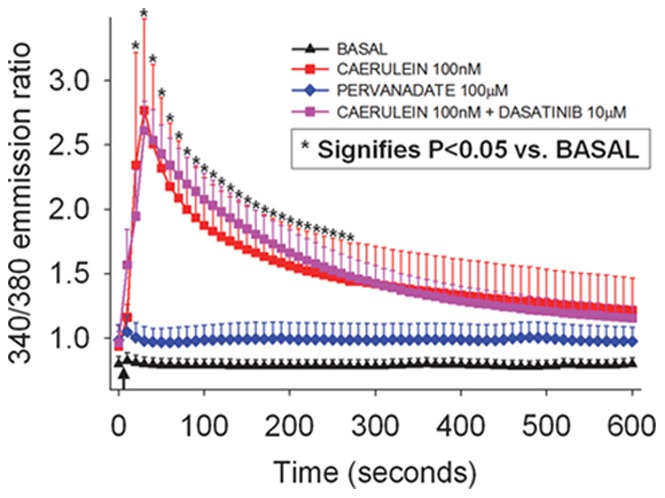
Pervanadate and Dasatinib do not affect resting or caerulein induced changes in cytosolic calcium. Cytosolic calcium levels in Fura-2AM loaded acini measured over 10 minutes. Arrow indicates time of addition of the stimulus. 100 µM pervanadate (PV, blue diamonds) does not result in an increase in resting cytosolic calcium levels, which remain unchanged in its presence, similar to basal acini (BASAL, black triangles). 100 nM Caerulein (100 nM CER, red squares), causes a prompt increase in cytosolic calcium levels, which is not reduced in the presence of 10 µM Dasatinib (100 nM CER+10 µM DAS, pink squares). Each data point represents mean value calculated over multiple (n≥3) experiments, in each of which 7–25 acini per field were analyzed. Standard error of mean is depicted as a bar. Asterisks correspond to time points at which calcium levels were significantly different (*p*<0.05) from basal levels compared to either conditions with 100 nM Caerulein alone, or to the same in the presence of 10 µM Dasatinib. Calcium levels in conditions with 100 µM Pervanadate were not significantly different from basal.

Since several studies have proposed an increase in cytosolic calcium to be necessary for premature trypsinogen activation [28]–[31], we therefore studied if pervanadate increases cytosolic calcium, or if Src inhibition with Dasatinib affects caerulein induced increases in cytosolic calcium. While there was no increase in cytosolic calcium under basal conditions over 10 minutes (black triangles, BASAL Figure 4), 100 nM caerulein caused a rapid and appropriate increase in cytosolic calcium (red squares). This caerulein induced increase was unaffected by Dasatinib, with which the acini had been pre-incubated and which was present in the medium throughout the assay (pink squares). Surprisingly, pervanadate (blue diamonds) did not cause an increase in cytosolic calcium after addition to acini over this duration, during which we note significant Src activation (Figure 4).

## Discussion

In this study we note that pervanadate treatment of acinar cells results in F-actin reorganization, trypsinogen activation and acinar injury independent of an increase in cytosolic calcium. This seems to involve Src since Dasatinib prevents the activation of Src, in addition to inhibiting F-actin reorganization, antegrade fragmentation of the Golgi, trypsinogen activation and acinar injury induced by pervanadate, which on its own does not increase cytosolic calcium levels. These phenomena induced by pervanadate are very similar to those known to be induced by supraphysiologic caerulein (apart from the increase in cytosolic calcium), the injurious effects of which were also prevented by Dasatinib.

These findings suggest the possibility of acinar cell injury being triggered by a complex involving aberrant vesicular trafficking and cytoskeletal events such as antegrade vesiculation of the Golgi [26] and reorganization of F-actin to the basolateral surface [7], [22] respectively, all without increasing cytosolic calcium concentrations. So far the ligands known to induce reorganization of actin in acinar cells via receptor mediated mechanism (e.g. carbamylcholine, caerulein at supraphysiologic concentrations) also increase cytosolic calcium [48]. While agents inducing oxidant stress such as hydrogen peroxide reorganize F-actin in acinar cells and induce cell injury, chelation of intracellular calcium has shown to prevent this and resulting acinar injury in the same study [49]. Similarly, studies using the intracellular calcium chelator BAPTA-AM [28], [50], [51], extracellular chelator EGTA [50], omission of calcium in the extracellular medium [28] have shown dependence of trypsinogen activation on calcium whether mediated by supraphysiologic caerulein or TNF-α [51]. Water immersion stress also reduced trypsinogen activation along with reducing basal levels as well as the peak and plateau cytosolic calcium levels induced by supraphysiologic caerulein [52]. However, the sole increase in cytosolic calcium such as with thapsigargin or ionomycin does not result in trypsinogen activation [28]. Therefore, the question whether it is the basal calcium levels necessary for cell signaling or the elevation in cytosolic calcium induced by injurious agents that plays a role in the deleterious outcomes is so far unanswered. Interestingly, supraphysiologic caerulein induced Src activation has been shown to be prevented by chelation of intracellular calcium [33]. Conversely, store mediated calcium entry has been thought to be Src dependent based on its inhibition using PP1 [53]. Our studies however show that Dasatinib did not inhibit the cytosolic calcium increase induced by caerulein. This seems logical, since the release of intracellular calcium induced by supraphysiologic caerulein peaks within a few seconds of its addition and likely precedes the activation of Src which peaks between 1–2 minutes [36].

In this study, the antibody used to immuno-precipitate and pull down Src binds an epitope common to all Src family members. We therefore have not characterized the specific member(s) involved in the phenomena noted. Acinar cells express several members of the Src family, including c-Src, Lyn, Yes, Fyn [7], [22], [36] which are activated by diverse stimuli [13], [36] and are proposed to have diverse functions [34]. It remains to be explored whether it is the magnitude of Src activation or the activation of specific members of the Src family that play a role in the outcomes we note. Supraphysiologic caerulein has been shown to activate both Yes and Lyn [7], [13], [36], which are the two best studied Src family members in acinar cells. However, both of these are partially activated by the high affinity CCK receptor agonist JMV 180 [13], [36], though to a lesser extent than supraphysiologic caerulein. JMV 180 however antagonizes low affinity receptor activation, does not result in trypsinogen activation [54], and reduces the severity of caerulein induced pancreatitis [55].

Potential members that have been implicated in post Golgi trafficking in other systems include c-Src [37]. The Golgi has been thought to regulate trypsinogen activation in acinar cells via pro-cathepsin B processing [26]. Yes has been thought to regulate actin dynamics in acinar cells [7], [22] and is involved in injurious blebbing. The activation of trypsin, followed by its secretion by agonists such as bombesin does not result in acinar injury [56]. Therefore the depletion of F-actin from the sub-apical surface as induced by supramaximal caerulein, and the prevention of this with partial restoration of secretion, as shown for PP2 [22], support the role of the induction of both trypsinogen activation and inhibition of secretion bring responsible for acinar injury. This is supported by recent data that carbamylcholine, which at high doses also activates Src family members [13] depletes subapical actin [48] and results in acinar injury [57]. While we do not propose that Src family kinase activation is solely responsible for the observed outcomes, the inhibition of these by Dasatinib, suggests the involvement of the Src family in the phenomena noted. Previous studies looking at vanadate have found it ineffective in preventing trypsinogen activation in acinar cells [58], which is in agreement with our data.

In summary, we have described two key cell biological events- antegrade vesiculation of the Golgi and apical F-actin depletion and its reorganization to the basolateral surface, which in previous studies have been shown to be involved in trypsinogen activation [26] and injurious blebbing of acinar cells [7] respectively, to result from an agent-pervanadate, without pervanadate elevating cytosolic calcium levels. This suggests that acinar cell injury can be initiated independent of an increase in cytosolic calcium. The inhibition of these phenomena and those induced by supraphysiologic caerulein by Src inhibition [7], [37] without Dasatinib affecting the increase in cytosolic calcium induced by caerulein support the need of further studies to explore the role of Src in these phenomena.
